# Additive manufacturing inert gas flow path strategies for multi-laser powder bed fusion systems and their impact on lattice structure mechanical responses

**DOI:** 10.1186/s41205-024-00212-3

**Published:** 2024-04-08

**Authors:** Sean P. Philips, Abigail Tetteh, Matthew A. Di Prima, Albert Burchi, Daniel A. Porter

**Affiliations:** 1 Division of Applied Mechanics, Office of Science and Engineering Laboratories, Center for Devices and Radiological Health, United States Food and Drug Administration, 10903 New Hampshire Ave., WO62-2215, MD 20993 USA; 2 EOS North America, Novi, MI 48377 USA

## Abstract

Multi-laser Additive Manufacturing systems hold great potential to increase productivity. However, adding multiple energy sources to a powder bed fusion system requires careful selection of a laser scan and inert gas flow strategy to optimize component performance. In this work, we explore four different laser scan and argon flow strategies on the quasi-static compressive mechanical response of Body Centered Cubic lattices. Three strategies employ a swim lane method where laser pathing tends to progress parallel to argon flow. Method one only uses a single laser while method two uses four, both with the laser path working against the argon flow. The third method uses four lasers, each operating in their own lane like the second method, but the laser pathing progresses with the argon flow. The fourth method has all four lasers operating in quadrants and the laser pathing trends against the argon flow.

The single-laser strategy generally had the lowest mechanical responses compared to the other three strategies. A quadrant strategy generally had the highest quasi-static mechanical responses and was at least 25% greater in stiffness, yield force, ultimate force, and energy absorption when compared to the single laser strategy. However, the four-laser swim strategy where the laser pathing tends against the argon flow was found to be statistically similar to the quadrant strategy. It is hypothesized that spatter introduced onto the powder layer from the melt pool and particle entrainment may be worse for laser pathing which trends with the argon flow direction. Additionally, the additional energy added to the build volume helps to mitigate inter-layer cool time which reduces temperature gradients. This shows that multi-laser AM systems have an impact on part performance and potentially shows lattices built with multi-laser AM systems may have certain advantages over single-laser AM systems.

## Introduction

Additive Manufacturing (AM) is a process of fabricating objects layer by layer from a simple or complex 3-dimensional model [1–7]. Per ISO/ASTM there are seven methods that fall under the umbrella of AM [8]. These different AM methods are used to produce parts that previously would have resulted in higher material waste, material usage, and energy consumption [1–3, 6]. AM can help in designing components quickly and efficiently [1, 2, 6]. As a rapidly developing technology, continued interest in product development appears to maintain momentum in the medical device area [9, 10]. Additionally, interest and usage of the AM technology persists in the aerospace and automotive fields.

Many AM medical devices utilize porous structures to help promote biological fixation [11, 12]. In 2016 about 67% of the ~ 80 AM medical devices cleared or approved by the U.S. FDA had some sort of porous structure [9]. By 2020, the number of cleared or approved AM medical devices increased to about 357 and the use of multi-laser AM systems was reported [10]. Additionally, most long-term implantable AM devices are metal and made using laser-powder bed fusion [9, 10]. Acetabular implants [13], spine cages [14], and chest wall implants [15] are a few examples of devices that fit into this category. With the recent trends in medical device clearances and approvals using additive manufacturing, it is anticipated that strategies to increase production and lower price per part will be employed. While adding more lasers may increase productivity for the fabrication of medical devices, performance alterations of the devices should be considered.

Laser Powder Bed Fusion (L-PBF) is an AM method that produces parts using powder and a laser energy source to thermally fuse the material together. Multi-Laser PBF (ML-PBF) is a L-PBF process, characterized by the simultaneous operation of multiple lasers to fuse the powder. This addition of multiple lasers may be beneficial as it increases build rates [16, 17] and there are reports of the potential to improve processing conditions such as temperature gradients and local cooling rates [16, 17]. ML-PBF production of parts is also coupled with a series of issues such as residual stress formed from temperature gradients and cooling inequalities, spatter, porosity, and lack of fusion that are prevalent in most AM methods.

Residual stress is a common adverse effect of L-PBF. This is due to the heating, cooling, and reheating of the material as well as temperature gradients within layers [4–6, 16–19]. The buildup of residual stresses can cause the formation of cracks, delamination and other forms of deformation that can negatively impact the mechanical properties of parts [4, 16, 18, 19]. The literature reports a significant increase in the residual stresses when multiple lasers are employed [7, 18]. However, using optimal laser scan strategies may reduce residual stress [19]. Temperature differences within and between layers of a part create residual stresses that can cause temperature gradients in the heat affected zone resulting in shrinkage [4, 6, 18, 19] and non-uniform cooling [17] which inevitably leads to buildup of residual stresses that can cause deformation. Zou et al. found that in certain in silico cases ML-PBF has the potential to reduce average stresses in the Ti-6Al-4 V print bed by 19% [18]. This reduction of residual stress is important when creating products with consistent mechanical and dimensional quality.

Spatter is a naturally occurring phenomenon in L-PBF and it is the source for many defects that can adversely impact the mechanical properties of the component being built [4, 20–24]. Literature reports that when introducing additional laser energy sources, more spatter is ejected from the powder bed by increasing the recoil pressure above the melt pools [21, 23]. Often Argon gas flow is used over the build plate to sweep away the spatter from the print bed [20, 23]. Because of the metal vapor plume created by evaporation of the powder, spatter can enter the flow of the protective gas in a process called entrainment [4, 20–22]. Ly et al. [22] reported that the majority of spatter is ejected in the direction that is opposite the laser path. Spatter causes print quality issues as it typically has a separate chemical makeup, and, due to coalescence, can be much larger than the feedstock powder [4, 23, 24]. The differing chemical compositions coupled with the larger physical size of the spatter coalescence can result in harder to melt particles or un-melted regions which result in inefficient use of laser energy. Additionally, these larger particles may exhibit balling [4]. The large chunks can be caught by the recoating arm which then creates voids and divots in the powder which further leads to inefficient use of the laser’s energy [21]. Thus, spatter causes many issues for L-PBF and potentially even more so for ML-PBF.

Porosity from gas being subsumed under the powder can cause vapor depression zones or keyholes. Lack of fusion is also prevalent when spatter is involved due to inclusions of un-melted particles. Balling of spatter can create regions lacking powder, which results in uneven melt pools, thus not being able to properly fuse the region. Porosity can initiate cracks and deformations within layers that inevitably leads to a degradation of mechanical properties within the part [4, 17]. Therefore, porosity may be higher if non-optimal gas flow strategies are used.

Post processing heat treatments are a common method [6] employed to alleviate residual stress [2, 16] within the part. To reduce degradation of mechanical properties from porosity, many metallic parts in L-PBF undergo thermal treatments such as hot isostatic pressing [25–27]. The goal of these heat treatments is to prevent warpage, cracks, and increase mechanical properties. However, post-process heat treatments generally cannot rectify cracks that are created during the build process [16]. Additionally, the treatments increase the time and cost of producing samples. Therefore, instead of only relying on thermal post processing to reduce porosity and residual stress, scan and airflow strategies can be employed to ameliorate temperature gradients, spatter, lack of fusion and other issues that cause detrimental part performance.

Scan strategies are predetermined methods that helps dictate how lasers traverse the build plate and melt powder. Scan strategies are crucial to ML-PBF as the amount of residual stress in a part is influenced by the chosen scan strategy [4, 5, 7, 17–20]. Many scan strategies have been developed and studied with the goal of mitigating the production of residual stress in manufactured parts. Zhang et al. investigated 12 different scan strategies to compare the effects of each strategy on temperature, final residual stresses, and z-direction deflections [19]. Additionally, He et al. describes “An Intelligent Scanning Strategy (SmartScan)” to achieve optimized uniform temperature distribution [16]. Despite the use of several scan strategies, large temperature gradients and spatter are unavoidable, but the goal of properly selecting a scan strategy, is to mitigate the formation of defects and residual stresses as much as possible. In addition to selecting a fitting scan strategy for a build, introducing a protective gas flow can also be beneficial.

This study employs four distinct combinations of laser scan and argon flow strategies on L-PBF Ti-6Al-4 V lattice structures to evaluate the differences in quasi-static mechanical responses. These lattice structures are of similar design and material to those used in medical devices. The efforts also vary the number of lasers used in the printing of samples to compare ML-PBF to single L-PBF. After each lattice sample was printed in one of four methods, they were subjected to a compression test to extract compressive mechanical performance metrics. A statistical analysis on the compressive metrics were performed to quantify potential differences. Additionally, μCT scans were performed on the samples to observe geometric differences. The results may help future users of multi-laser AM systems more efficiently select a process setup that caters to their device performance needs.

## Materials and methods

**Fig. 1:**
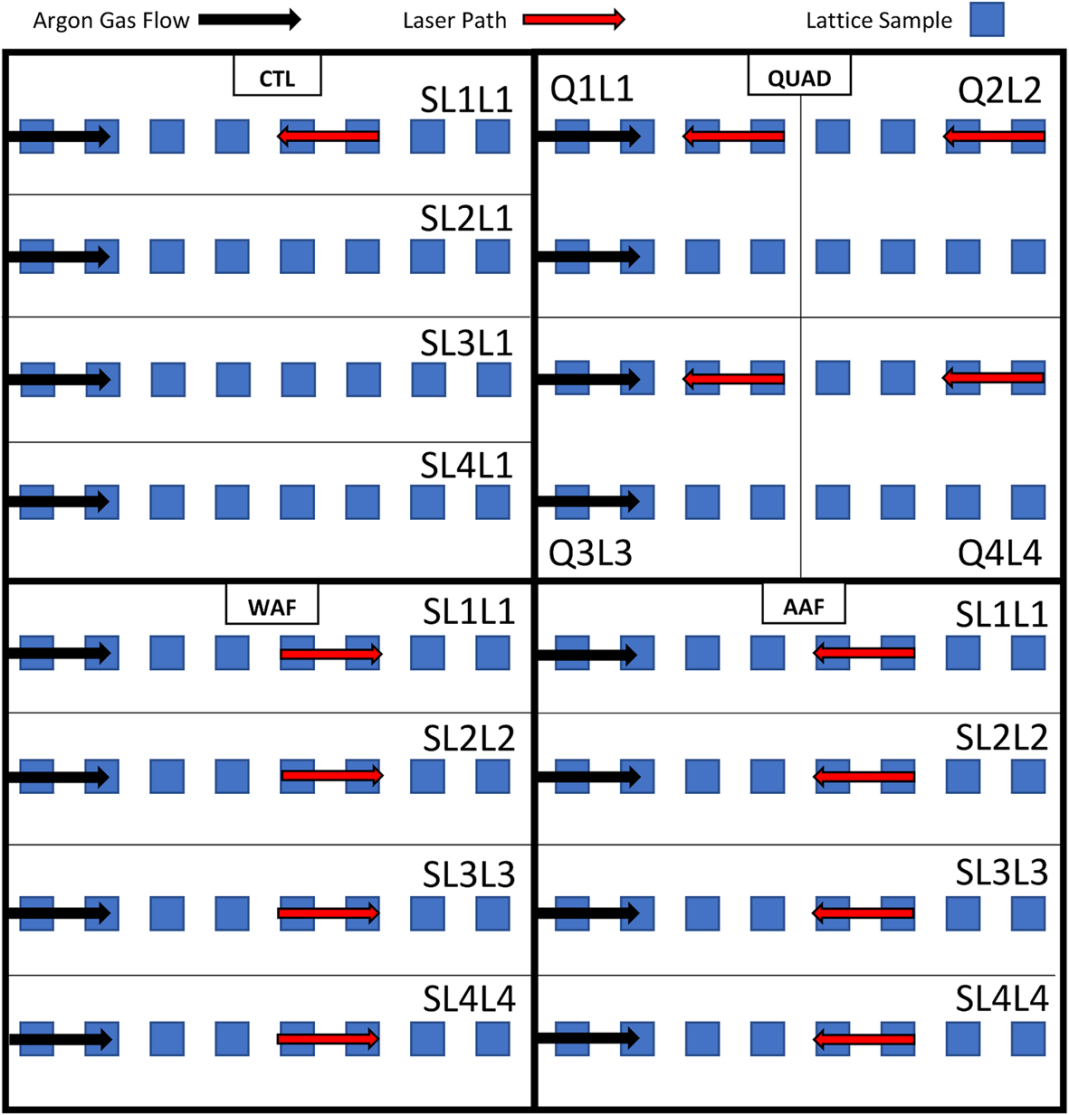
Print methods for the four strategies which are CTL, QUAD, WAF, and AAF. Q1L1 signifies Quadrant 1 Laser 1 and SL1L1 indicates Swim Lane 1 Laser 1. Argon gas flow direction and the trending path of the laser is marked by black and red arrows respectively. *n* = 32 per strategy

A preliminary study was performed to help identify a sample design which printed well given default print parameters and was representative of medical device lattice structures. Regular-repeating Body Centered Cubic (BCC) lattice structures made from Ti-6Al-4 V were selected from the preliminary study. Samples were approximately 12 mm in diameter, 29.5 mm in height, and had a designed strut diameter of 0.4 mm and a pore size of 1.3 mm. All samples were fabricated from Ti-6Al-4 V powder on an EOS M300–4 (Electro Optical Systems GmbH; Krailling, Germany). This AM system employs an argon flow sheath from left to right over the build plate (when facing the primary window of the system). Four different laser scan and argon flow strategies were employed, Fig. 1. Nominal print parameters from the OEM’s software for the AM system were used since they generally resulted in reasonable starting mechanical outputs.

One of the strategies organized the samples into quadrants while the other three used “swim lanes” which ensures that parts are organized into four isolated horizontal rows. The first strategy is the control group (CTL) where only one laser was used for all swim lanes and the laser scan direction going against the argon flow. This counter gas flow-laser path strategy is considered the traditional method as it causes the spatter to be deposited on part surfaces which have already been processed by the laser. A second strategy used the quadrant method (QUAD) where all four lasers worked in their individual quadrants, but also employing a counter gas flow-laser path strategy. It was assumed that a control group for the QUAD strategy was not needed since the argon flow interactions in the single-laser CTL swim lane strategy would be similar to the argon flow interaction of the single-laser quadrant strategy. The third strategy used all four lasers in their isolated swim lanes but instead of traditional laser-gas pathing, had the laser path direction going with the argon flow (WAF). Lastly, the fourth strategy again used all four lasers in their isolated swim lanes and utilized the traditional laser-gas pathing strategy of the laser path direction going against argon flow (AAF). 32 lattice samples were fabricated for each strategy. The number of replicates was chosen to avoid overpacking the build plate and so that the location of samples did not have to be altered (geometric symmetry) to accommodate the four different strategies.

**Fig. 2:**
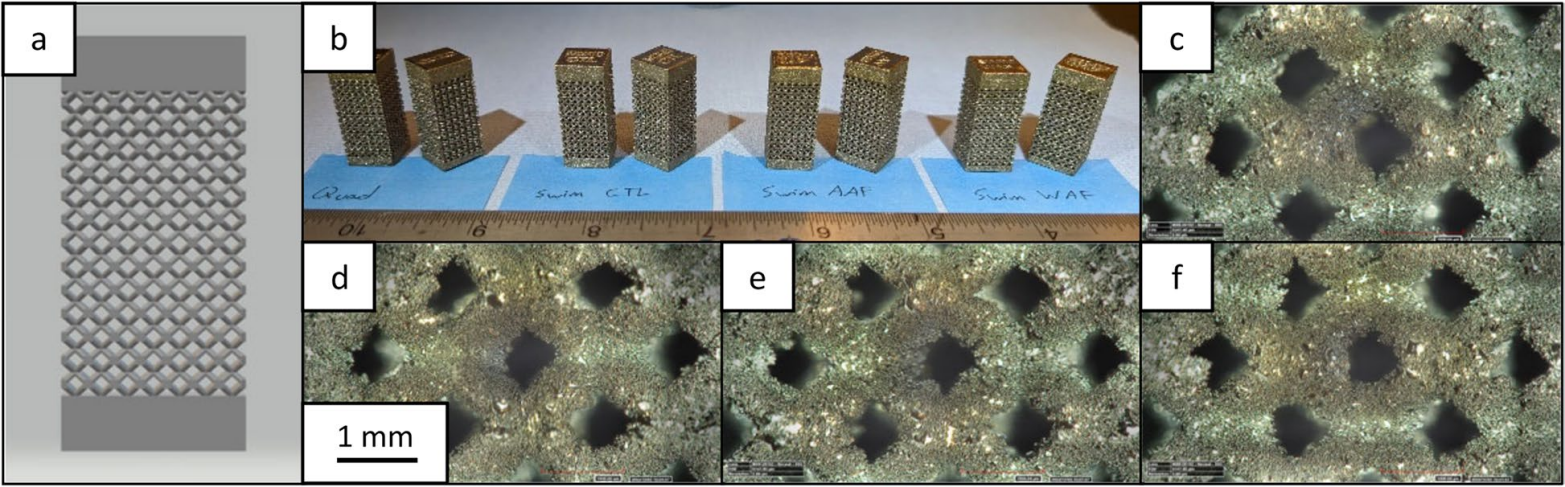
Images of the lattice samples. **a** The STL imported into the EOS software and **b** each sample type lined up. 60x magnification images of a **c** CTL, **d** WAF, **e** AAF, and **f** QUAD sample

Before compression testing, optical imaging was conducted on multiple samples of each print strategy group using a Hirox RH-2000 microscope with an MXB-2016Z Zoom Lens and RH-2000 Ver 2.0.40 software (Hirox Co Ltd.; Tokyo, Japan). The images, Fig. 2, indicate no immediate discernable differences between sample types.

### Compression testing

After optical microscopy was completed, lattice samples were marked and numbered from 1 to 30 for each strategy type. Two samples from each strategy build were arbitrarily reserved for μCT scanning. To minimize sequential testing order bias error, each sample was then randomized using an Excel document to dictate compression testing order. Lattice samples were then subjected to quasi-static compression testing using a 68FM-100,100 kN load frame (Instron; Norwood, MA) that was equipped with hardened stainless-steel platens. Additional steel shims were inserted below and above the sample to protect the platens from permanent damage potentially caused from the Ti-6Al-4 V lattice structures. During testing, the load frame was set to compress at a crosshead displacement rate of 6 mm per minute with a cutoff at 10 mm of total crosshead displacement. Many samples were not subjected to the full 10 mm displacement as there was obvious failure before the cutoff displacement. The reaction force and LVDT displacement of the platens were acquired from Bluehill software (Instron; Norwood, Massachusetts). A webcam captured video of each sample as it underwent the compression testing.

After data collection, all samples were analyzed using a python script to extract stiffness, 0.2% yield, ultimate strength, compression displacement at break, and energy capacity to break. A One-Way ANOVA with post-hoc Tukey test were conducted using Minitab 17 software (Minitab LLC; State College, PA).

### Micro-CT scanning

Micro-computed tomography (μCT) was used to determine the mean strut thickness, pore size, and relative density for two lattice samples per strategy. This may reveal potential dimensional differences between the laser and gas strategies. All lattices were scanned with a Scanco Medical μCT100 (Scanco Medical, Brüttisellen, Switzerland). The samples were placed in 19 mm diameter by 84 mm height tubes and used a 0.1 mm Cu filter. Scanning parameters were 90 kVp voltage, 200 μA beam current, and 350 ms integration time. The scan resolution was set to ‘high’ with an isotropic voxel resolution of 7.4 μm. Scanning time of each specimen was 62.7 minutes for 645 axial slices.

The Bone Trabecular Morphometry algorithm within the Scanco μCT program was used to estimate geometric metrics of each lattice. Contours were drawn manually, and a fixed threshold was applied to segment struts from pore spacing. The evaluation area for each sample was ~ 0.95 cm^2^ and 0.74 cm high. Afterwards, the morphometric evaluation program that implements Direct Transformation (DT) mapping was used to quantify the average strut thickness, pore size, and relative density values of each sample.

## Results

**Fig. 3:**
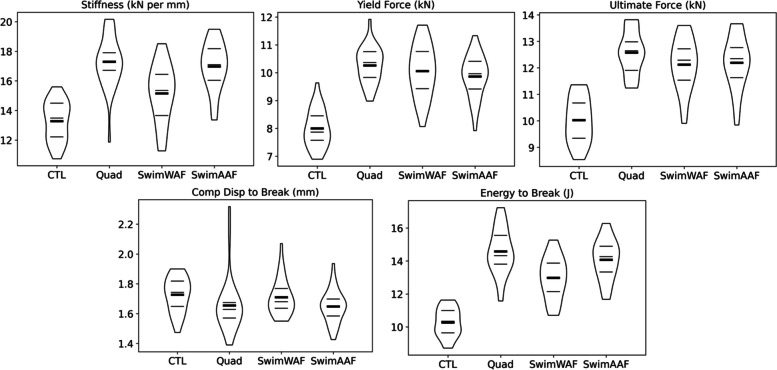
Violin plots for lattice sample stiffness (kN/mm), 0.2% yield (kN), ultimate force (kN), compressive displacement to break (mm), and energy to break (J). The thin horizontal lines are the 1st, 2nd, and 3rd quartiles while the thick horizontal line is the sample mean. *n* = 30 per strategy

Quasi-static compression performance metrics for all four multi-laser strategies is shown in Fig. 3. Differences can be observed immediately in the printing strategies. Breaking down the data quantitatively further shows discrepancies between sample groups. The CTL samples have the lowest mean for all metric categories except for the compressive displacement to break. On average, QUAD samples were 30.3% stiffer, yielded at forces 28.3% larger, had 25.5% greater ultimate forces, and were able to absorb 42.0% more energy than the CTL strategy. Although the CTL samples had the lowest stiffness, they did have the highest average displacement at break of any group on average. The second lowest performing strategy appeared to be the WAF. This was due to it being 14.1% stiffer, having 20.9% greater ultimate forces, and absorbing 26.4% more energy than the CTL strategy.

A post-ANOVA Tukey analysis revealed that many of the lattice compression performance metrics were statistically different, Table 3 (Appendix). For ease of review, a compact letter display revealing statistical similarity groupings (on means, *p* < 0.05, Tukey) is shown in Table 1. Except for displacement at break, all other metrics means had at least one strategy which was statistically different. The most similar groups appeared to be the QUAD and AAF. The AAF samples had 1.9, 3.9, 3.1, 0.4, and 3.4% lower stiffness, yield force, ultimate force, displacement at break, and energy at break, respectively, then the samples in the QUAD group. Interestingly, AAF and WAF sample groups had similar yield strength (*p* = 0.756) and ultimate strength (*p* = 0.990) but differed much more in stiffness and energy absorption (*p* = 0.000 and *p* = 0.003 respectively). The AAF samples were 12.1% stiffer and absorbed 8.5% more energy than WAF samples before break. It is important to note is that the cool down times between each layer for the multi-laser groups was about 10 seconds compared to 25 seconds for the single-laser CTL group. This could account for some of the mechanical performance differences observed and highlights the increased build speed of using multiple lasers.

**Table 1:** Statistics and similarity groupings for all four laser-gas strategies and five compressive performance metrics, *n* = 30 per strategy. Groups with common letter identifiers (e.g., A) were found to be statistically similar (*p* < 0.05, Tukey)

	Mean	St. Dev.	Grouping
Stiffness (kN/mm)			
QUAD	17.30	1.73	A
AAF	16.98	1.61	A
WAF	15.15	1.96	B
CTL	13.28	1.33	C
Yield Force (kN)			
QUAD	10.26	0.65	A
AAF	9.86	0.76	A
WAF	10.06	0.94	A
CTL	8.00	0.67	B
Ultimate Force (kN)			
QUAD	12.58	0.83	A
AAF	12.19	1.00	A
WAF	12.12	0.97	A
CTL	10.03	0.86	B
Disp. at Break (mm)			
QUAD	1.66	0.18	A
AAF	1.65	0.11	A
WAF	1.71	0.12	A
CTL	1.73	0.12	A
Energy to Break (J)			
QUAD	14.58	1.40	A
AAF	14.08	1.22	A
WAF	12.98	1.27	B
CTL	10.27	0.85	C

**Fig. 4:**
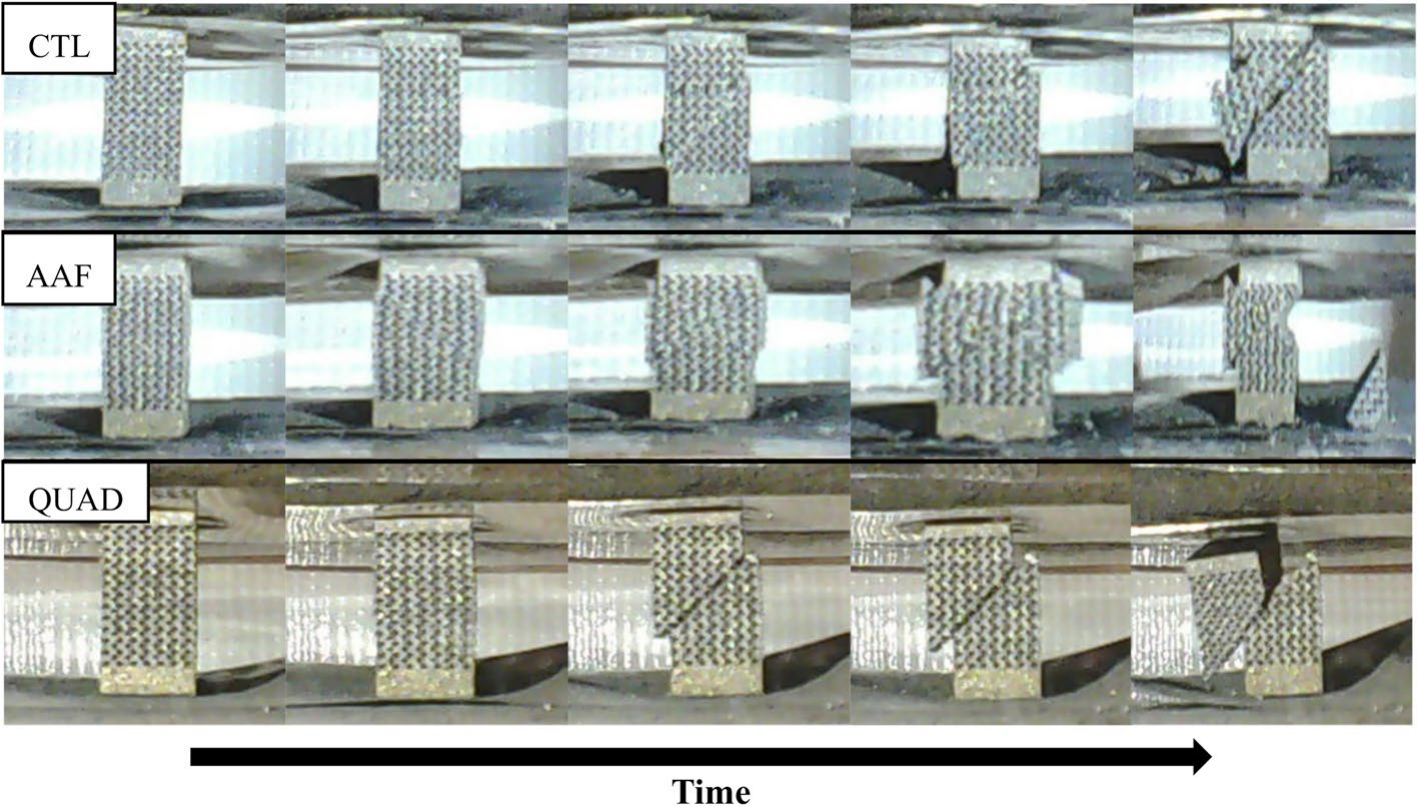
Failure modes shown in a series of time lapse images for three samples undergoing compression. Images depict CTL sample #6, AAF sample #20, and QUAD sample #26. Failures such as Quad sample #26 were the most common

Three types of failure modes that were observed during sample compression testing are shown in Fig. 4. The failure shown in CTL sample #6 sheared along a single plain, like most samples, but also had a chunk break off due to multiple fractures. The AAF sample #20 and QUAD sample #16 were the only lattices which suffered from double shear failure and resulted in two triangular chunks breaking away. This event was rare and only occurred twice out of the 120 tested samples. No samples in the CTL or WAF groups failed in this manner. The most common failure mode that occurred in almost every sample, was shearing of a single plane. This either occurred in a controlled manner or resulted in a high energy release fracture resulting in two larger pieces. This controlled manner of shearing in one plane can be seen clearly in Fig. 4 where QUAD sample #26 underwent compression, sheared, and the top part was slowly pushed down the slope created by the shear. The samples that kinetically exploded, either were completely ejected off the platens or only left one of the larger halves behind.

**Fig. 5:**
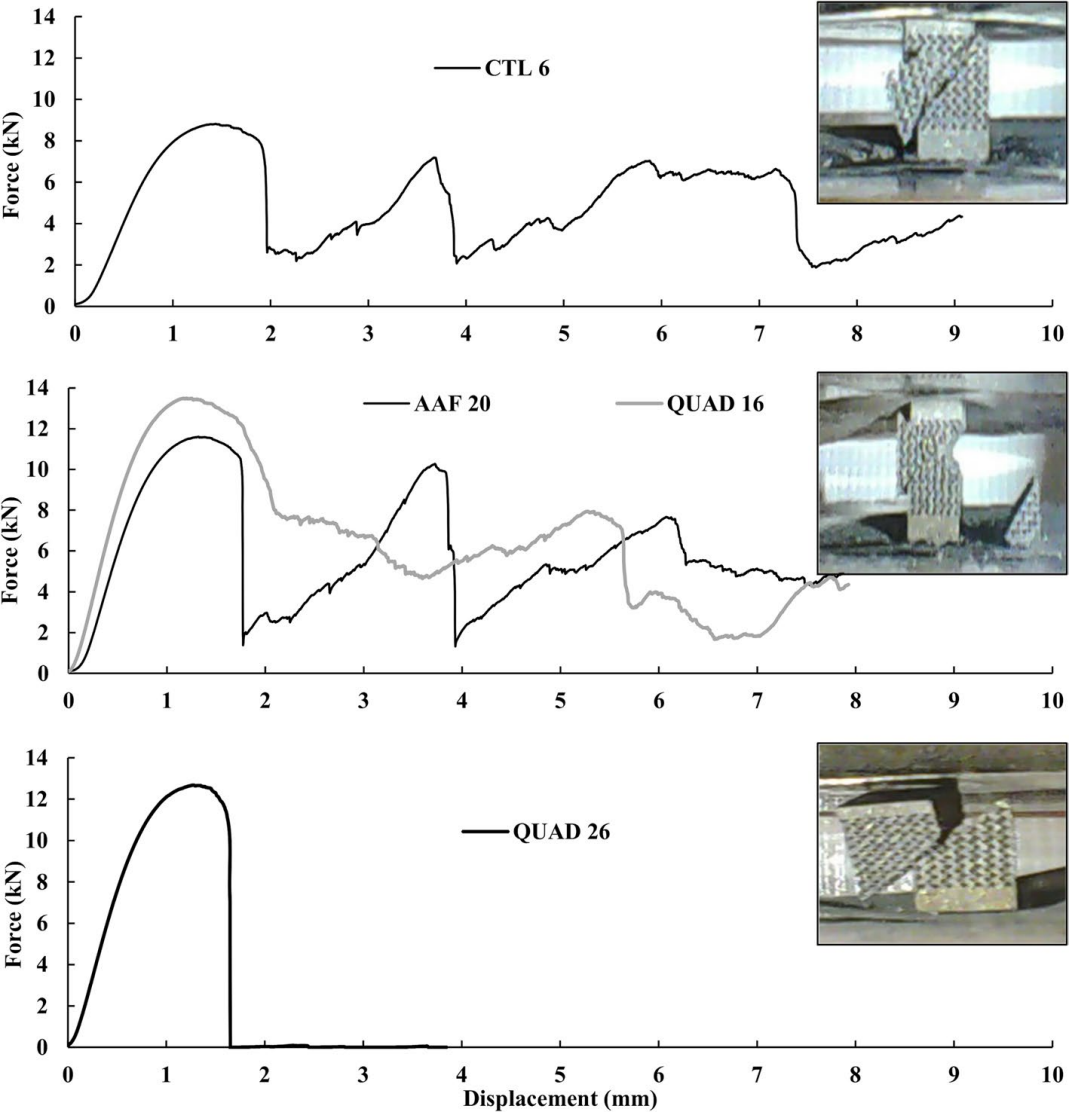
Load-displacement behavior of the three typical failure modes observed in the lattice specimens during compression testing

Load-displacement curves of the identified samples in Fig. 4 are shown in Fig. 5. Every dip in the applied force is a point where a significant strut fracture occurs. The graph for QUAD 26 shows the most common occurrence in the sample testing. Generally, samples quickly reached their ultimate strength then fractured and sheared apart. Some of these samples had graphs which extended further when portions of the lattice structure were caught as shearing and sliding occurred. However, the principal force drop-off after ultimate force remained constant. In the other images, the samples also have that same drop-off but also show multiple peaks that are the results of multiple shearing and other fracture events. For example, the second large peak that almost reaches the force of the first peak in the AAF 20 load displacement curve, is the second shear that creates the final hourglass cutout for that lattice sample.

### Micro CT results

Average values of strut thickness, pore size, and relative density for all four laser and gas strategies is shown in Table 2. Using the Bone Trabecular Morphometry algorithm in the Scanco software, differences between geometric parameters appears small with the percent differences (relative to the QUAD parameters) being under 4%. A meaningful standard deviation could not be calculated from only two samples per strategy. Instead, spread was measured as the max difference between any of the two samples with results of 0.011 mm, 0.013 mm, and 0.033 for the strut thickness, pore size, and relative density. It is interesting to note that these max differences all occurred in the CTL strategy and were at least 100% higher in the strut thickness and pore size category and at least 50% higher in the relative density category than any other strategy. This may indicate that the CTL strategy could induce higher lattice dimensional variability.

**Table 2:** Average μCT results for the different laser and gas flow strategies. *n* = 2 per strategy

	Strut Thickness			Pore Size			Relative Density		
	Mean (mm)	Range (mm)	% Diff^*^	Mean (mm)	Range (mm)	% Diff^*^	Mean	Range	% Diff^*^
QUAD	0.288	0.003		0.679	0.004		0.329	0.010	
AAF	0.293	0.003	1.6	0.685	0.004	0.9	0.330	0.013	0.3
WAF	0.296	0.005	2.7	0.688	0.003	1.3	0.321	0.021	−2.4
CTL	0.296	0.011	2.7	0.678	0.013	−0.2	0.319	0.033	−3.1

^*^Relative to QUAD sample group

## Discussion

It is evident that the single laser CTL group generally has statistically lower compressive mechanical properties compared to the other print strategies. The QUAD and AAF group were the strongest on average as they exhibited the highest metrics in four categories: stiffness, yield force, ultimate strength, and energy at break. The differences in mechanical properties between the four sample groups can potentially be attributed to three factors: the number of lasers, the scan strategy, and the gas flow direction.

The discrepancy between the CTL sample group and the rest of the samples can be greatly attributed to the fact that the CTL lot was fabricated using one laser. With only a single laser, there was much more time for layers to cool before the re-coater arm applied more powder and the laser swept through to melt that newly laid powder. Although the samples were printed in swim lanes like the WAF and AAF samples, the single laser strategy took about four times longer to scan the powder layer. This delay between melting of layers results in higher temperature gradients between the current and previous layers. In QUAD, WAF, and AAF print strategies, each swim lane is printed simultaneously allowing for less cooling time. Any cooling from the flow of argon gas would also be experienced more by CTL parts. It has been shown that a single laser generally results in lower peak temperatures within the build chamber compared to multiple lasers [5, 19]. Therefore, less lasers used would create a larger temperature gradient between the melt pools and the surrounding environment. The increased cooling experienced by CTL samples is a reasonable explanation for the lower mechanical properties observed.

The laser scan strategy employed for WAF and AAF samples used four parallel swim lanes and four lasers. QUAD also used four lasers but in a quadrant scan strategy. Literature indicates the residual stress of a part may rely heavily on the scan pattern/strategy. An island based scan strategy may reduce residual stress in fabricated parts [17] due to a reduction of temperature gradients within the build. It has been reported that larger islands may result in more residual stress while more islands may result in less stress [5, 17]. Due to the partitioning of the build plate into quarters for both QUAD and swim lane strategies, the overall area of the islands remain the same, yet the perimeters differ. With the QUAD strategy, all partitions have equal perimeter ratios and may be more efficient at reducing temperature gradients when compared to the other scan strategies. The scan strategy of WAF and AAF sample groups were similar (just opposite direction) and thus likely created similar residual stress.

Argon gas flowed in the same direction for each build strategy. This is important because it always flowed from left to right when looking into the build chamber. The WAF sample group had an alternate laser scan strategy that appeared to have caused more issues in the build compared to its counterpart AAF. Due to the WAF laser scan strategy, the laser generally progressed in the direction of the argon gas flow. Argon gas flow sweeps the ejected spatter particles away from the lasers immediate working area. As explained in [22], the spatter generated by the laser mostly ejects in the direction of entrainment which for the WAF samples opposed the flow of the argon gas. Therefore, potentially resulting in a situation similar to [20] where many spatter particles may land in the path of the laser on the print. Spatter is highly detrimental to the mechanical properties of ML-PBF parts and WAF samples were potentially more susceptible to the effects of spatter as the shielding argon gas flow was rendered less effective.

As ML-PBF and AM fabrication grows more prevalent, there is a growing need for finding a build strategy that yields consistent parts with strong mechanical properties. Through the literature review, many different scan strategies were proposed such as the “Intelligent Scan Strategy (SmartScan)” [16]. Another method observed in multiple articles was a two-laser delayed strategy which uses two lasers to scan the same area with a slight time delay between scans and was found effective at lowering residual stress [7, 16, 18, 19]. It’s anticipated to be due to the second laser working to keep the temperature of the melt pool area higher, reducing the speed at which the cooling occurs [18, 19].

Future studies could investigate compressive fatigue performance of the four different strategies and determine if similar trends exist. It would be interesting to see if the detrimental effect of spatter is amplified under fatigue loading. Another beneficial investigation could be to test multiple builds and encompass the effect of powder reuse on the observations in this work. Investigations into potential grain structure and phase differences would also be beneficial in uncovering root causes of the mechanical property differences; however, it could also be expected that the mechanical observations in the multi-laser groups may lead one to believe that there would be minimal difference in microstructure.

Given the current data in this study, altering laser and gas flow strategy can have a significant effect on quasi-static mechanical properties. Therefore, re-validation should be carefully considered when altering the laser and gas flow strategy for a currently manufactured medical device [28]. This is due to the possibility of static mechanical performance of components changing and no longer meeting pre-determined specifications.

## Conclusion

Medical devices fabricated using single-laser AM systems may need revalidation if transitioning to multi-laser AM systems. From the lattice compression test observations, important differences can be distinguished between the laser and gas flow strategy sample groups. Lattices built with multi-laser AM systems may have a quasi-static mechanical property advantage over single laser AM systems. The QUAD strategy samples, on average, had the largest quasi-static mechanical responses as they exhibited the highest stiffness, yield force, ultimate force, and energy to break. The QUAD mean values for these metrics were 30.3, 28.3, 25.5, and 42.0% greater than those of the single laser CTL means respectively. AAF samples were the next strongest on average. AAF and QUAD mean mechanical property metrics were within 4% difference between each other. The only strategy difference between WAF and AAF scan methods, is the direction in which the lasers scanned. The WAF laser scan strategy was the only method to generally traverse with the argon flow. This discrepancy appeared to render WAF samples with the third highest strength based on stiffness and energy absorption. On average, the AAF was 12.1% stiffer and absorbed 8.5% more energy to break than the WAF samples. Scan strategy had no appreciable effect on the mean dimensions of the lattice strut thickness, pore size, and relative density. However, the CTL group had significantly more variability (as measured by range) in each of the three-dimensional categories. Despite the single laser CTL strategy being the overall weakest sample group, all four strategies did have a statistically similar compressive displacement at break.

## Data Availability

Data may be made available upon reasonable request.

## Appendix

**Table 3:** Post-ANOVA Tukey analysis comparing inter-strategy means for lattice compressive performance metrics. *p* < 0.05, *n* = 30 per strategy

Adj. *P*-Value	WAF-CTL	AAF-CTL	QUAD-CTL	AAF-WAF	QUAD-WAF	QUAD-AAF
Stiffness (kN/mm)	0.000	0.000	0.000	0.000	0.000	0.882
0.2% Yield Force (kN)	0.000	0.000	0.000	0.756	0.736	0.192
Ultimate Force (kN)	0.000	0.000	0.000	0.990	0.224	0.374
Disp at Break (mm)	0.960	0.113	0.176	0.295	0.406	0.997
Energy to Break (J)	0.000	0.000	0.000	0.003	0.000	0.384
